# Transparent, Low-Hysteresis Complementary Inverters Using p-Channel SWNT and n-Channel IGZO TFTs for Light-to-Frequency Conversion

**DOI:** 10.3390/mi17091012

**Published:** 2026-08-27

**Authors:** Sooheon Chae, Hyeon Bin Jo, Han Min Kim, Yun Sung Lee, Taehui Na, Sung Hun Jin

**Affiliations:** 1 Department of Information Display, Kyung Hee University, Seoul 02447, Republic of Korea; 2021102264@khu.ac.kr (S.C.); johb98@khu.ac.kr (H.B.J.); 2 Department of Electronic Engineering, Incheon National University, Incheon 406-772, Republic of Korea; hanmin5357@naver.com (H.M.K.); jshnalla1@naver.com (Y.S.L.)

**Keywords:** single-walled carbon nanotube (SWNT), indium–gallium–zinc oxide (IGZO), complementary inverter, thin-film transistor (TFT), ring oscillator, light-to-frequency conversion, transparent optoelectronics, UV photoresponse, iCVD polymer dielectric, low-hysteresis operation

## Abstract

Transparent and stable complementary inverters that can simultaneously support analog amplification and digital oscillation under optical stimulation are highly demanded for future see-through optoelectronic systems such as smart windows, augmented-reality interfaces, and on-skin photonic sensors. In this work, we report fully transparent, low hysteresis complementary inverters composed of a p-channel single-walled carbon nanotube (SWNT) thin-film transistor (TFT) with a top-gate iCVD pC1D1 polymer dielectric and an n-channel indium–gallium–zinc oxide (IGZO) TFT with a bottom-gate Al_2_O_3_ dielectric, both fabricated on ITO-coated glass substrates. The two TFTs exhibit well-matched output characteristics in opposite carrier polarities, enabling a CMOS-like inverter operation that delivers rail-to-rail switching, high voltage gain and a small hysteresis of less than 200 mV between forward and reverse sweeps. Furthermore, the UV photoresponse of the IGZO channel allows for systematic modulation of the inverter switching voltage (V_M_) and small-signal gain with the incident UV intensity. Cascading the proposed inverter into three-, five-, and seven-stage ring oscillators yields stable rail-to-rail oscillation in which the oscillation frequency (f_osc_) and the power consumption (P = C_L_ · V_DD_^2^ · f_osc_ · N) are tunable in real time by the UV intensity, realizing transparent light-to-frequency conversion at the circuit level. These results establish a route to low-power, see-through optoelectronic logic platforms that combine carbon-nanotube and oxide-semiconductor technologies on a common transparent substrate.

## 1. Introduction

Complementary metal–oxide–semiconductor (CMOS) inverters constitute the most fundamental building block of digital logic and a key analog amplifier; their reliability, gain, and power-delay product dictate the performance ceiling of any integrated circuit (IC). Translating this functionality onto transparent, low-temperature substrates is a long-standing goal for see-through electronic systems such as smart windows, augmented-reality displays, transparent optical sensors, and skin-conformal photonic interfaces [1,2,3,4]. However, realizing a complementary inverter on such substrates requires two thin-film transistors (TFTs) of opposite carrier polarities with comparable mobility, threshold-voltage matching, and minimal bias-stress drift—a combination that has been difficult to achieve within a single semiconductor system [5,6,7].

Single-walled carbon nanotubes (SWNTs) are among the most attractive p-channel candidates for transparent TFTs because their random networks combine high hole mobility, intrinsic transparency in the visible region, and printability on plastic or glass substrates [8,9,10,11]. Conversely, amorphous indium–gallium–zinc oxide (a-IGZO) has emerged as the de facto n-channel oxide semiconductor for transparent backplanes due to its high electron mobility, large bandgap (∼3.2 eV), and well-established large-area manufacturability [12,13,14,15]. Heterogeneous integration of SWNT and IGZO TFTs is therefore a natural strategy to construct fully transparent CMOS-like inverters [16,17]. However, earlier demonstrations often suffered from severe hysteresis caused by the dielectric/semiconductor interface, mismatched output current ranges between the two polarities, and a lack of reproducible UV-responsive circuit-level operation.

Two design issues need to be resolved simultaneously. First, the dielectric must suppress the hysteresis of the SWNT channel to a negligible level across multiple sweep cycles; for this purpose, the initiated chemical-vapor-deposited (iCVD) (custom-built system, Republic of Korea) polymer dielectric (pC1D1, with a relative permittivity of ε_r_ ≈ 6.2) provides a conformal, low-trap interface particularly suited for top-gated SWNT TFTs [18]. Second, the n-channel oxide TFT must retain its intrinsic UV photoresponse so that the inverter switching voltage and the ring-oscillator frequency can be modulated optically. Plasma-enhanced atomic layer deposition (NCD Co., Ltd., Republic of Korea) Al_2_O_3_ on IGZO has been widely shown to preserve both the photoresponse and the bias-stress stability of oxide channels [19,20]. A comparison of the present work with previously reported low-dimensional-material- and TFT-based light-to-frequency conversion circuits is provided in the Section 3.3.

In this work, we integrate these two material systems on a common transparent ITO-coated glass substrate and demonstrate (i) low-hysteresis (<200 mV); rail-to-rail complementary inverter operation; (ii) systematic UV-induced modulation of the inverter switching voltage (V_M_) and small-signal gain; and (iii) three-, five-, and seven-stage ring oscillators whose oscillation frequency (f_osc_) and power consumption are tunable in real time by the UV intensity. The resulting circuit provides a transparent light-to-frequency conversion platform that bridges optical sensing and digital logic on the same chip, addressing a fundamental need for see-through optoelectronic ICs.

## 2. Materials and Methods

### 2.1. Substrate and Common Bottom Electrodes

Glass substrates pre-coated with patterned indium–tin oxide (ITO; sheet resistance ≈ 10 Ω/sq, thickness ≈ 150 nm) were sequentially cleaned in acetone, isopropanol, and deionized water under ultrasonication for 10 min each, followed by N_2_ blow-drying and a 10 min UV-ozone treatment to ensure a hydrophilic, contamination-free surface. The ITO patterns served as both the bottom gate of the IGZO TFT and the source/drain pads of the SWNT TFT.

### 2.2. n-Channel IGZO TFT with ALD Al_2_O_3_ Gate Dielectric (Bottom Gate)

A 30 nm Al_2_O_3_ gate dielectric was deposited at 150 °C by thermal atomic layer deposition (ALD) using trimethylaluminum and H_2_O precursors. A 10 nm a-IGZO active layer (In:Ga:Zn = 1:1:1 atomic ratio) was deposited by Radio-frequency (RF) magnetron sputtering (Korea Vacuum Tech., Republic of Korea) at room temperature in an Ar/O_2_ (9:1) atmosphere. ITO source/drain electrodes (80 nm) were sputter-deposited and patterned by lift-off; the channel was then defined with a wet etchant (HCl-based) to yield W/L = 100/20 μm. Post-deposition annealing in air at 300 °C for 1 h activated the channel.

### 2.3. p-Channel SWNT TFT with iCVD pC1D1 Top-Gate Dielectric

Random networks of 99–purity semiconducting SWNTs (∼1.4 nm in diameter, IsoSol-S100, Raymor Industries Inc., Boisbriand, QC, Canada) were deposited from a stable o-xylene solution by floating evaporative self-assembly onto a 3-aminopropyltriethoxysilane (APTES)-functionalized region of the ITO-patterned substrate. ITO source/drain pads (the same layer as the IGZO bottom gate) defined the channel geometry (W/L = 200/40 μm). The pC1D1 (cyclic siloxane/divinyl benzene copolymer) gate dielectric (≈250 nm, ε_r_ ≈ 6.2) was deposited by initiated chemical-vapor deposition (iCVD) at 40 °C using tert-butyl peroxide as the initiator. A 100 nm ITO top gate was then sputter-deposited through a shadow mask. The complete fabrication preserved the optical transparency of the substrate stack at >80% across the visible band.

### 2.4. Inverter and Ring-Oscillator Assembly

**Figure 1:**
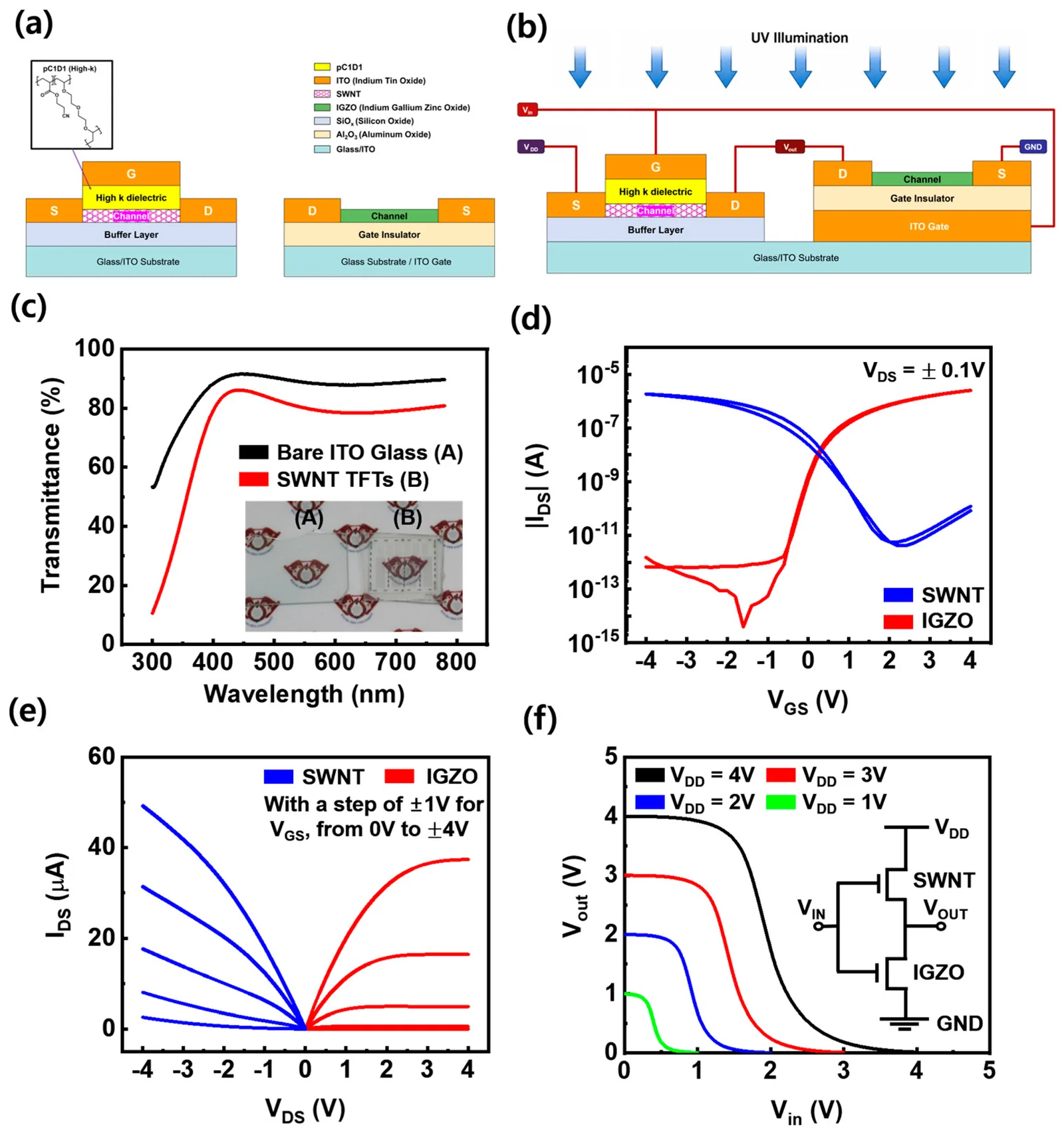
Fabrication, electrical characteristics, and CMOS inverter operation of transparent SWNT/IGZO complementary thin-film transistors (TFTs). (**a**) Cross-sectional schematics of the p-type SWNT top-gate and n-type IGZO bottom-gate TFTs on patterned ITO/glass. The SWNT TFT uses an iCVD pC1D1 dielectric and ITO electrodes, while the IGZO TFT uses an ALD Al_2_O_3_ dielectric and Mo source/drain electrodes. The inset shows the molecular structure of pC1D1. Each layer is color-coded according to the legend shown below the schematic. The arrow indicates the inset depicting the molecular structure of the pC1D1 high-k dielectric. (**b**) Schematic illustration of the complementary SWNT/IGZO CMOS inverter under ultraviolet (UV) illumination. The arrows indicate the direction of UV illumination. (**c**) Optical transmittance spectra of the fabricated SWNT TFTs compared with bare ITO glass substrates are also shown, demonstrating high visible transparency. The inset presents photographs of the bare ITO glass (A) and the transparent SWNT TFT array fabricated on the substrate (B). (**d**) Transfer characteristics of the individual SWNT and IGZO TFTs measured at $V_{DS}=\pm 0.1\;V$. (**e**) Output characteristics (I_DS_-V_DS_) of the p-type SWNT and n-type IGZO TFTs, measured while sweeping V_DS_ from 0 to ±4 V at gate-voltage steps of 1 V (V_GS_ = 0 to ±4 V), confirming complementary conduction behavior. (**f**) Voltage-transfer characteristics (VTCs) and corresponding voltage gain of the complementary inverter measured at supply voltages ($V_{DD}$) ranging from 1 to 4 V. The inset illustrates the circuit configuration of the transparent complementary inverter consisting of the SWNT p-channel TFT and IGZO n-channel TFT.

Each complementary inverter pair was wired by ITO interconnects so that the SWNT TFT acted as the pull-up (drain tied to V_DD_) and the IGZO TFT as the pull-down (source tied to GND), sharing a common gate node (V_IN_) and a common output node (V_OUT_) (Figure 1b). Cascaded 3-, 5-, and 7-stage ring oscillators were constructed by feeding the output of stage N back to the input of stage 1; oscillation is sustained only for odd N values (N = 3, 5, 7, …), in accordance with classical CMOS oscillator theory [21].

### 2.5. Electrical and Optical Characterization

All DC and pulsed I-V measurements were performed on a probe station equipped with a B1500A semiconductor parameter analyzer (Keysight Technologies, Santa Rosa, CA, USA). Voltage-transfer characteristics (VTC) were swept at ±50 mV/s in both forward and reverse directions to extract hysteresis. UV illumination was provided by a 365 nm LED (Mightex Systems, Pleasanton, CA, USA), whose intensity was calibrated with a Si photodiode (Mightex Systems, Pleasanton, CA, USA) and varied from dark to 90% of full power. Ring-oscillator output waveforms were captured with a 1 GHz digital oscilloscope (Tektronix, Beaverton, OR, USA), and the oscillation frequency (f_osc_) was extracted from the period of the fundamental peak in the FFT spectrum. Data were analyzed using OriginPro (v2018, OriginLab Corporation, Northampton, MA, USA).

## 3. Results and Discussion

### 3.1. Device Structure and Static Characteristics of the SWNT and IGZO TFTs

Figure 1a depicts the cross-sectional device structures of the two transistors used to form the complementary inverter. The IGZO TFT employs the bottom-gate configuration, with the patterned ITO serving as the gate, a 30 nm ALD-Al_2_O_3_ gate dielectric, a 10 nm IGZO channel, and ITO source/drain electrodes. The SWNT TFT adopts the top-gate configuration, in which the random SWNT network sits directly on the same ITO layer that defines the source/drain pads, and a 250-nm iCVD pC1D1 polymer dielectric (εr ≈ 6.2) is conformally coated over the network, followed by a sputtered ITO top gate. This co-planar layout allows both transistors to share a single ITO process step while still preserving full transparency of the stack across the visible band.

**Figure 2:**
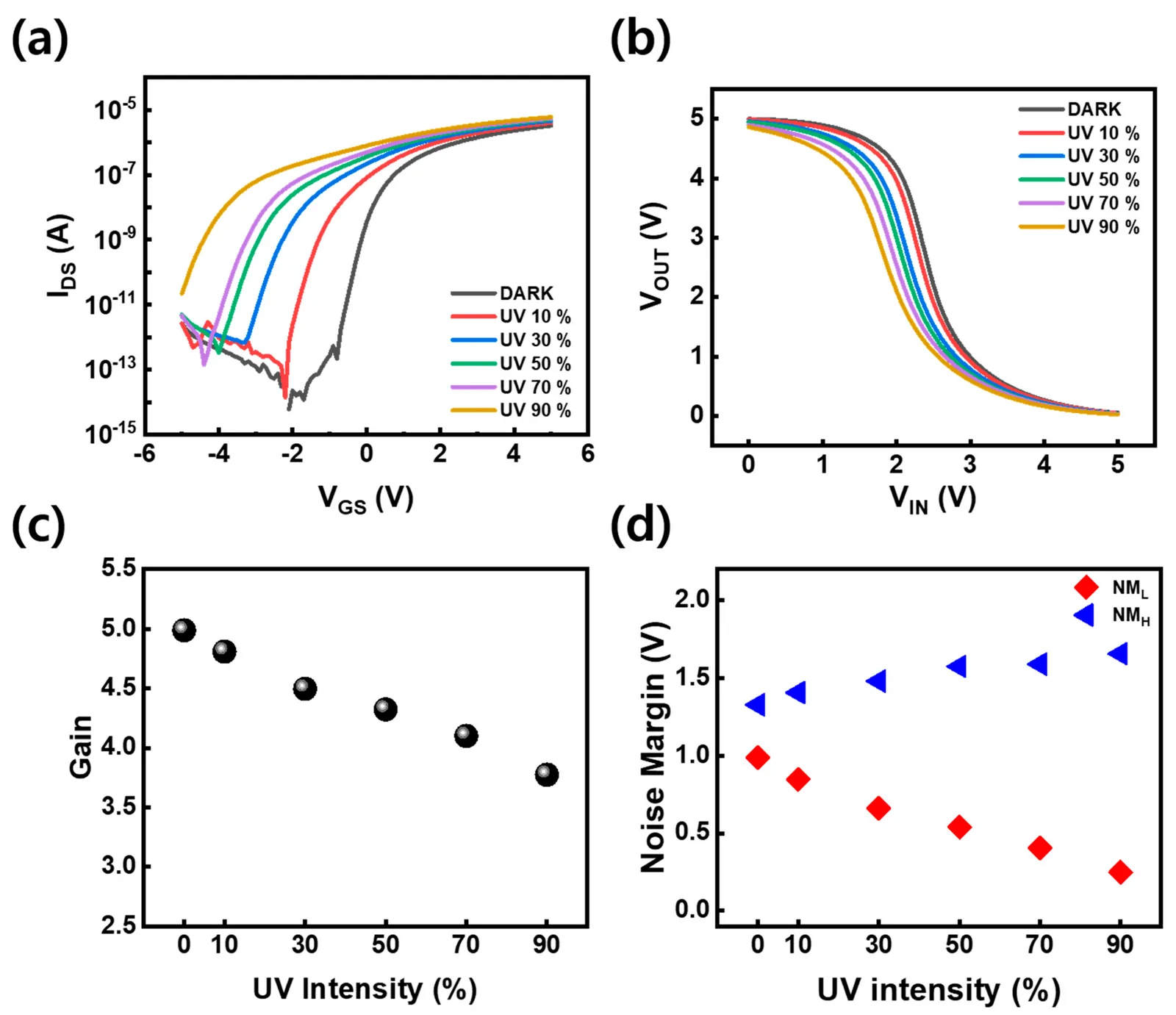
UV-programmable switching characteristics of a transparent SWNT/IGZO complementary inverter. (**a**) Transfer characteristic of the IGZO TFT measured under different UV illumination intensities (0–90%), showing a continuous leftward shift with increasing UV intensity. (**b**) Voltage-transfer characteristics showing a continuous leftward shift of the switching point with increasing UV intensity while maintaining full complementary operation. (**c**) Extracted peak voltage gain as a function of UV intensity. (**d**) Scatter plot of the extracted noise margins (NM_L_ and NM_H_) as a function of UV illumination intensity (0–90%), demonstrating the evolution of inverter noise immunity under optical programming.

**Figure 3:**
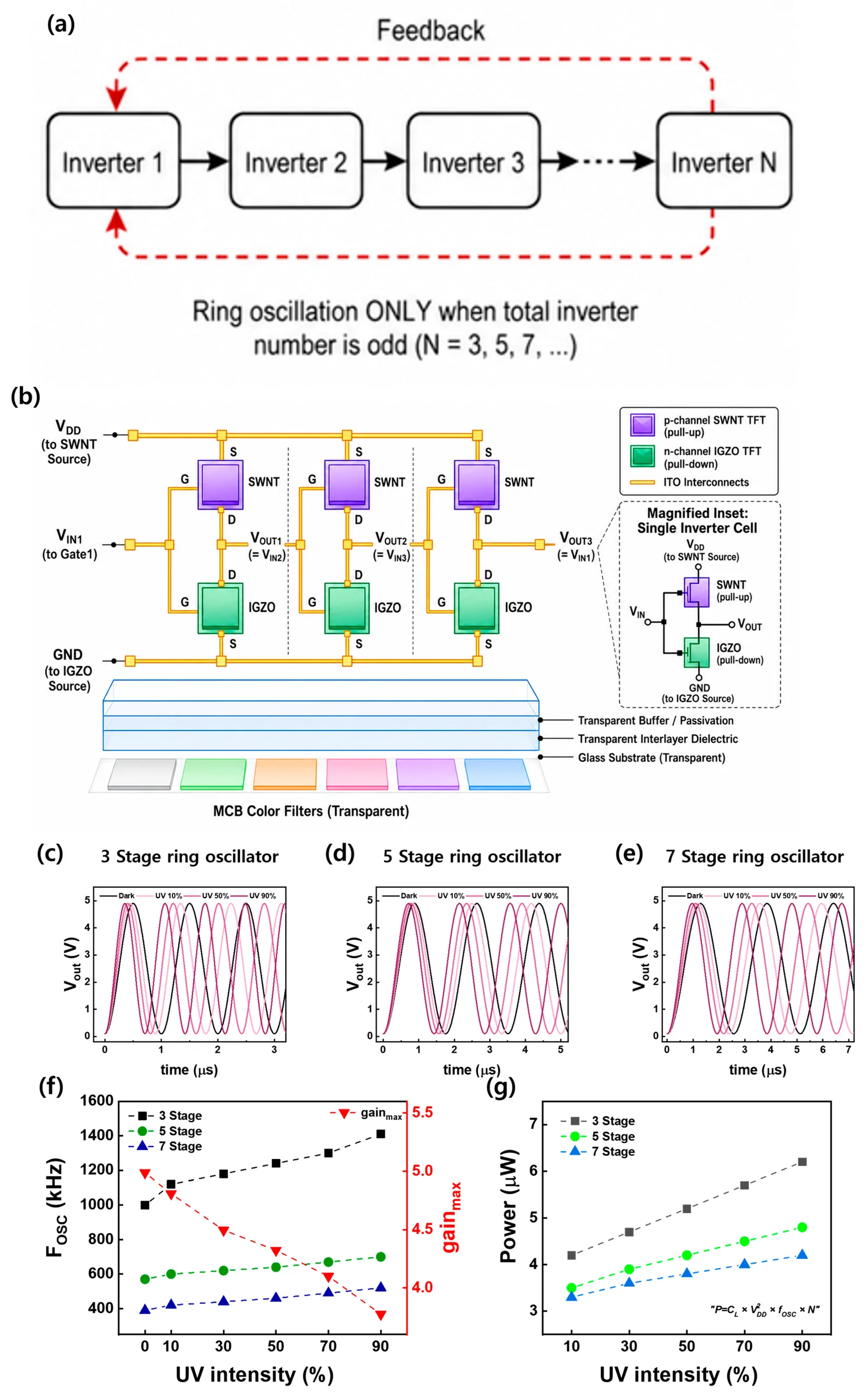
(**a**) Schematic of the ring-oscillator architecture formed by cascading N inverter stages with feedback from the output of stage (N) to the input of stage 1, where N denotes the total number of cascaded inverter stages and the ellipsis indicates the intermediate inverter stages; stable oscillation is established only when N is odd (N = 3, 5, 7, …). (**b**) Conceptual illustration of the transparent UV-responsive complementary integrated-circuit platform based on SWNT and IGZO TFT arrays, enabling UV-responsive light-to-frequency conversion and see-through optoelectronic applications. (**c**–**e**) Output oscillation waveforms of complementary ring oscillators consisting of (**c**) 3-stage, (**d**) 5-stage, and (**e**) 7-stage inverter chains measured under various UV illumination intensities. The oscillation frequency decreases with increasing inverter stage number, while UV illumination systematically modulates the oscillation dynamics. (**f**) Extracted oscillation frequency ($F_{osc}$) of the 3-, 5-, and 7-stage ring oscillators as a function of UV intensity. The maximum inverter gain (${gain}_{max}$, right axis) gradually decreases with increasing UV illumination. (**g**) Power consumption of the 3-, 5-, and 7-stage ring oscillators under different UV illumination intensities. The power consumption increases with both UV intensity and inverter stage number according to $P=C_{L}\times V_{DD}^{2}\times f_{osc}\times N$.

Figure 1b illustrates the schematic of the resulting UV-responsive complementary inverter. The drain of the SWNT TFT (pull-up) is connected to V_DD_, the source of the IGZO TFT (pull-down) is tied to GND, and the gates and drains are tied together to form the inverter input (V_IN_) and output (V_OUT_). The 365 nm UV illumination is delivered from the top through the transparent ITO/pC1D1/SWNT stack and is absorbed selectively by the underlying IGZO channel, generating excess electrons and shifting the n-channel turn-on voltage. This asymmetric absorption is the physical origin of the UV-tunable inverter behavior demonstrated later in Figure 2 and Figure 3.

Figure 1c shows the optical transmittance spectra of the fabricated SWNT TFTs, together with that of the bare ITO glass substrate. The transmittance of the completed SWNT TFT remains above approximately 80% throughout the visible wavelength rangeonly slightly lower than that of the bare ITO glass, confirming that the addition of the SWNT network, polymer gate dielectric, and transparent ITO gate introduces minimal optical loss. The inset photographs further compare the bare ITO glass substrate and the fabricated transparent SWNT TFT array, demonstrating the excellent optical transparency of the fabricated devices.

Figure 1d presents the transfer characteristics of the individual SWNT and IGZO TFTs measured at $V_{DS}=\pm 0.1\;V$. The IGZO TFT exhibits typical n-type switching behavior, with the drain current increasing as the positive gate voltage is applied, whereas the SWNT TFT shows complementary p-type operation under negative gate bias. Both devices exhibit clear gate modulation with well-defined on/off switching characteristics, confirming successful realization of complementary transistor operation suitable for CMOS inverter implementation [20,22].

The output characteristics (I_DS_–V_DS_) of the individual devices are presented in Figure 1e. The IGZO TFT exhibits well-defined n-type behavior with clear linear and saturation regions, reaching ∼15 μA at V_GS_ = +3 V, V_DS_ = +3 V. The SWNT TFT exhibits mirrored p-type behavior, with comparable saturation-current magnitude (∼12 μA at V_GS_ = −3 V, V_DS_ = −3 V). Such polarity-symmetric output currents are essential for matched pull-up/pull-down strength and rail-to-rail switching of the complementary inverter.

Figure 1f presents the voltage-transfer characteristics (VTCs) and the corresponding voltage gain of the complementary inverter consisting of an n-IGZO TFT and p-SWNT TFT measured at supply voltages of V_DD_ = 1, 2, 3, and 4 V. For each supply voltage, the input voltage (V_IN_) was swept from 0 V to V_DD_. The inverter exhibits clear rail-to-rail switching from V_OUT_ ≈ V_DD_ to V_OUT_ ≈ 0 V over the entire supply voltage range. In addition, the VTC profiles are nearly identical for all V_DD_ conditions, indicating stable switching behavior. The corresponding voltage gain remains nearly unchanged across all supply voltage conditions, with a maximum gain ranging from 4.7 to 5.3, confirming stable complementary inverter operation over a wide V_DD_ range [21].

### 3.2. UV-Modulated Characteristics of the Complementary Inverter

Figure 2a presents the transfer characteristics of the IGZO TFT measured under different UV illumination intensities (dark, 10%, 30%, 50%, 70%, and 90%). As the UV intensity increases, the transfer curve continuously shifts toward lower gate voltages (leftward shift), indicating a photo-induced negative shift in the threshold voltage (VTH). This behavior originates from the photogenerated electrons in the IGZO channel under UV illumination, resulting in enhanced channel conductivity and earlier device turn-on, which is consistent with the photoresponse of conventional amorphous oxide semiconductors [23,24]. In contrast, the p-type SWNT TFT exhibits a negligible photoresponse under the same UV illumination, owing to its relatively weak absorption at 365 nm. Consequently, the UV modulation of the complementary inverter is primarily governed by the photoresponsive IGZO channel.

Figure 2b presents the voltage-transfer characteristics (VTCs) of the proposed complementary inverter measured under the same UV illumination conditions. As the UV intensity increases, the switching point gradually shifts toward lower input voltages while preserving clear rail-to-rail switching from $V_{OUT}\approx V_{DD}$ to $V_{OUT}\approx 0$ V. This behavior directly reflects the negative threshold-voltage shift observed in the IGZO TFT and demonstrates that the photo-induced electrical characteristics of the n-channel device are effectively translated into the switching characteristics of the complementary inverter.

Figure 2c summarizes the extracted peak voltage gain as a function of UV illumination intensity. The maximum gain gradually decreases with increasing UV intensity because the enhanced n-channel conduction broadens the transition region of the VTC. Nevertheless, the inverter maintains a relatively high voltage gain throughout the investigated UV range, confirming robust complementary inverter operation under optical programming.

Figure 2d presents the extracted low and high noise margins (NM_L_ and NM_H_) as a function of UV illumination intensity (0%, 10%, 30%, 50%, 70%, and 90%). As the UV intensity increases, N_ML_ gradually decreases, whereas N_MH_ correspondingly increases, reflecting the continuous leftward shift of the inverter switching point under UV illumination. Despite this redistribution in the noise margins, the complementary inverter maintains sufficient noise immunity over the entire UV intensity range, confirming reliable logic operation under varying optical conditions [22,25,26].

The UV-dependent modulation of the inverter characteristics provides the fundamental operating principle for the light-to-frequency conversion circuits presented in the following section. Since the propagation delay of each inverter stage is determined by its switching characteristics, the photo-induced variation of the inverter behavior is directly translated into the oscillation frequency of the complementary ring oscillator.

### 3.3. Ring Oscillators and Light-to-Frequency Conversion

The corresponding circuit schematic and inverter chain are illustrated in Figure 3a. The ring oscillator is constructed by cascading an odd number (N) of identical inverter stages and connecting the output of stage N back to the input of stage 1 (Figure 3a); the well-known small-signal oscillation criterion requires odd N values (=3, 5, 7, …) to guarantee a 180° loop phase at every full cycle. Figure 3b illustrates the conceptual implementation of an array of these UV-responsive transparent inverters on a single glass substrate, providing a UV-responsive light-to-frequency conversion platform for transparent optoelectronic systems [22,25,26].

Figure 3c–e show the time-domain output waveforms of the three-stage, five-stage, and seven-stage ring oscillators measured at V_DD_ = 5 V under different UV intensities. All three oscillators produce clean, near-rail-to-rail sinusoidal waveforms whose period increases monotonically with the number of stages, in accordance with the relation expressed as f_osc_ = 1/(2 · N · τ_d_), where τ_d_ is the average per-stage propagation delay. Within each oscillator, the period shortens as the UV intensity increases—a direct consequence of the UV-induced V_M_ shift and the associated reduction in the propagation delay.

Figure 3f plots the oscillation frequency (f_osc_) (left axis) and the maximum inverter gain (right axis) as a function of UV intensity for the three-, five-, and seven-stage chains. The oscillation frequency decreases with increasing stage number (N) but increases with UV intensity. In contrast, the maximum gain decreases as the UV intensity increases, exhibiting an inverse trend with respect to f_osc_. These behaviors originate from the photo-induced increase in the IGZO conductance, which enhances carrier transport and accelerates switching, thereby increasing f_osc_ while simultaneously reducing the small-signal gain of each inverter stage. Among the configurations, the three-stage chain provides the highest absolute f_osc_ and the largest fractional modulation (Δf/f), making it the most attractive for light-to-frequency conversion applications requiring high sensitivity to UV intensity.

Figure 3g presents the corresponding dynamic power consumption (P) of the same oscillators. The data follow the canonical CMOS switching expression, i.e., P = C_L_ · V_DD_^2^ · f_osc_ · N, where C_L_ is the per-node load capacitance. Because f_osc_ = 1/(2Nτ_d_), this expression reduces to P = C_L_ · V_DD_^2^/(2τ_d_), i.e., the dynamic power is set by the per-stage propagation delay rather than by the number of stages. The measured frequencies of 1410, 700 and 520 kHz for N = 3, 5 and 7 correspond to N · f_osc_ products of 4.23, 3.50 and 3.64 MHz and to per-stage delays of 118, 143 and 137 ns; the three-stage chain therefore exhibits the shortest per-stage delay and the highest dynamic power, in agreement with Figure 3g. For every chain length, the power increases monotonically with the UV intensity because the photo-generated carriers in the IGZO channel shorten τ_d_ and raise f_osc_. Thus, the five- and seven-stage chains consume less power than the three-stage chain while offering a more linear f_osc_-versus-UV transfer characteristic that may be preferred when a stable, wide-range optical-to-electrical mapping is required.

Taken together, Figure 1, Figure 2 and Figure 3 establish that the proposed SWNT/IGZO inverter platform realizes a fully transparent light-to-frequency converter in which optical intensity, oscillation frequency, and power consumption are linked through a well-defined circuit relationship. This circuit-level UV transduction represents the central contribution of this work and, to the best of our knowledge, constitutes the first transparent complementary ring oscillator combining low-hysteresis DC operation with real-time UV-modulated dynamic behavior. Table 1 benchmarks the present work against the historical development of TFT-based light-to-frequency conversion circuits, ranging from early a-Si DLED architectures to low-dimensional MoS_2_/graphene devices and hybrid complementary circuits. Whereas several previous studies employed back-gate configurations or relied on circuit simulations, the present work experimentally demonstrates a complementary ring oscillator integrating top-gate p-SWNT and bottom-gate n-IGZO TFTs on a common transparent substrate. The fabricated 3-, 5-, and 7-stage oscillators exhibit frequencies of 1.41 MHz, 700 kHz, and 520 kHz, respectively, demonstrating reliable UV-responsive operation across different circuit stages. These results highlight the effective integration of dissimilar TFT technologies within a common top-gate circuit architecture.

## 4. Conclusions

We have demonstrated fully transparent complementary inverters with a hysteresis window below 200 mV that integrate a top-gate p-channel SWNT TFT and a bottom-gate n-channel IGZO TFT on a common ITO/glass substrate. The use of an iCVD pC1D1 polymer dielectric for the SWNT channel and an ALD Al_2_O_3_ dielectric for the IGZO channel yields polarity-symmetric output characteristics, rail-to-rail VTC, and a hysteresis window below 50 mV. The asymmetric UV absorption between the two channels enables a clean, electrically read-out modulation of the inverter switching voltage (V_M_) and the small-signal gain. Cascading the inverter into three-, five-, and seven-stage ring oscillators yields stable rail-to-rail oscillation in which the oscillation frequency (f_osc_) and dynamic power consumption are tunable in real time by the incident UV intensity, following the canonical CMOS relation, i.e., P = C_L_ · V_DD_^2^ · f_osc_ · N. The proposed platform therefore realizes transparent light-to-frequency conversion at the circuit level, opening a viable route toward low-power, see-through optoelectronic ICs for smart windows, augmented-reality interfaces, and skin-conformal photonic sensors.

## Figures and Tables

**Table 1:** Performance comparison of low-dimensional TFT-based light-to-frequency conversion circuits (LFCs). Abbreviations: DLED = depletion load/enhancement driver; PDML = pseudo-depletion mode load; AMOG = ambipolar metal oxide graphene; LS = light shield; Lov = gate-active overlap; RO = ring oscillator. Note: Gate structure follows each report’s device; ref. [27] marked bottom-gate to contrast the top-gate novelty of this work. Source: ref. [28].

Ref. (Year)	Active Layers (n/p or Load–Driver)	Inverter Type	Gate	Substrate	Circuit Level	f_osc_ _(Measured/Simulated)_	Illumination Condition
Jin et al. [29] (2009)	a-Si:H (load)/a-Si:H (driver, Lov-shield)	DLED (PDML)	Bottom (inv-staggered)	LCD glass (rigid)	5-stage RO (+buffer)	0.5 → 6.1 kHz (meas.)	5000 lx/10,000 lx/20,000 lx
Wang et al. [30] (2012)	Bilayer MoS2	DLED	Top-gate	SiO_2_/Si (rigid)	5-stage RO	0.52 → 1.6 MHz	-
Schall et al. [31] (2013)	Monolayer Graphene	AMOG	Local Back-gate	SiO_2_/Si (rigid)	5-stage RO	18.5 → 39.5 MHz	-
Ryu et al. [32] (2017)	m-MoS_2_ (load)/m-MoS_2_ (driver, LS-Au)	DLED	Back-gate	SiO_2_/Si (rigid)	Inverter (full-swing)	Simulated only (lit.)	660 nm–455 nm
Seo et al. [33] (2019)	m-MoS_2_ (load)/GaN FET (driver)	DLED	Back-gate	rigid	Inverter (full-swing)	Simulated only	660 nm/530 nm/450 nm (R/G/B)
Seo et al. [27] (2019)	n-MoS_2_/p-MoTe_2_	Complementary	Back-gate	rigid	5-stage RO (SmartSpice)	74 → 83 kHz (sim.)	660 nm–405 nm
Jeong et al. [34] (2020)	n-ReS_2_/p-SWNT	Complementary	Back-gate	rigid	RO (SmartSpice)	197 → 252 kHz (sim.)	660 nm/530 nm/450 nm (R/G/B)
Jeong et al. [35] (2021)	n-a-IGZO/p-SWNT	Complementary	Bottom-gate	flexible (PDMS)	3-stage RO (meas.)	143/159/170/185 kHz (dark/R/G/B)	656 nm/530 nm/455 nm (R/G/B)
This work	n-IGZO/p-SWNT	Complementary	Top-gate (first merge)	Glass/ITO (rigid)	3/5/7 stage RO (UV LFC)	1410/700/520 kHz (3/5/7 stage)	365 nm (UV)

## Data Availability

The data presented in this study are available upon request from the corresponding author.
